# Determining firms׳ utility functions and competitive roles from data on market shares using Lotka–Volterra models

**DOI:** 10.1016/j.dib.2016.03.020

**Published:** 2016-03-10

**Authors:** A. Marasco, A. Picucci, A. Romano

**Affiliations:** a Department of Mathematics and Applications, University of Naples Federico II, Via Cintia, 80126 Naples, Italy; b Via P. Della Valle, 80126 Naples, Italy; c Erasmus University, Rotterdam Institute of Law and Economics (RILE), 3062 PA Rotterdam, The Netherlands

## Abstract

In this article, we include data on historical and estimated market shares of two markets. In particular, we include annual data on the market shares of the Japanese beer market (1963–2000) and biannual data on the market shares of the mobile phones market in Greece (1998–2007). In addition, we estimate monthly data on market shares for both markets. We show how this data can be used to derive firms’ utility functions and their competitive roles.

**Specifications Table**

**Table:** 

Subject area	*Economics, Business*
More specific subject area	*Planning and Forecasting in Business*
Type of data	*Table*
How data was acquired	*The market shares of the three main Japanese beer companies are taken from*[1]*. The market shares of three mobile phone companies in Greece are taken from*[2]*. The estimated market shares are derived from the model.*
Data format	*Raw*
Experimental factors	*N/A*
Experimental features	*We analyze the data using a nonoautonomous integrable Lotka Volterra model to: (i) derive the utility functions of the firms, (ii) estimate their market shares, and (iii) determine firms’ competitive roles.*
Data source location	*Greece, Japan*
Data accessibility	*Data with this article*

**Value of the data**

• Accurate and reliable historical data on market shares is only seldom available. These historical series (38 and 19 data, respectively) are very valuable because they cover a relatively long time horizon and they refer to markets that are widely recognized as relevant.
• We estimate a series of infra-annual (monthly) data points to offer a clearer picture of the dynamics of the two markets (445 and 109 data, respectively). The real and the estimated data can be used to compare the dynamics of these markets with the dynamics of markets of the same products in other countries.
• The data on market shares allow to determine firms׳ utility functions and their competitive roles.

## 1 Data

**Fig. 1:**
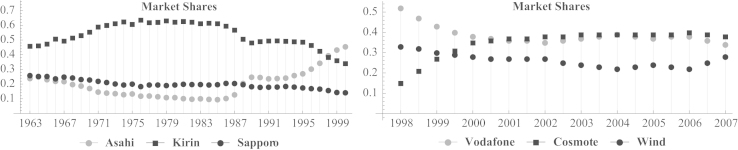
Market shares of three Japanese beer companies (left panel), and of three mobile phone companies in Greece (right panel).

**Fig. 2:**
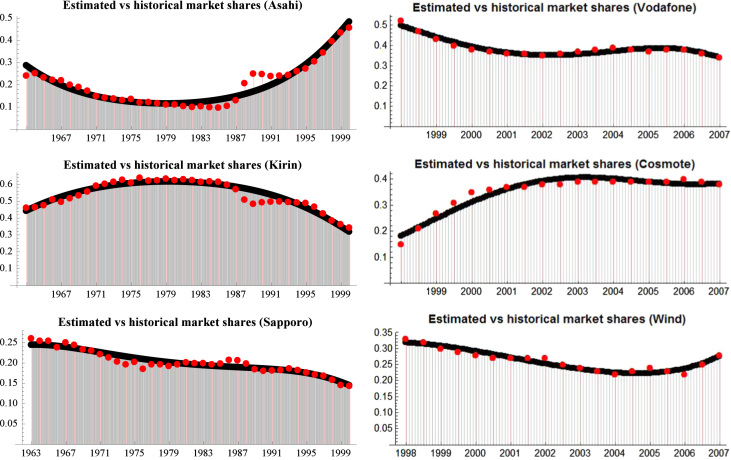
Estimated (black points) versus historical market shares (red points) of three Japanese beer companies (left panel), and of three mobile phone companies in Greece (right panel).

We include a historical series of market shares for two markets (see Fig. 1 and refer to Supplementary materials). More precisely, we present the market shares of the beer market in Japan and the mobile phone market in Greece. For the former, we have annual observations from 1963 to 2000. For the latter, we have biannual observations from 1998 to 2007. Moreover, we estimate infra-annual data of market shares for both market (see Fig. 2 and refer to Supplementary materials).

## 2 Experimental design, materials and methods

### 2.1 Experimental design

The data on the Japanese beer market only regards the three main beer producers, whereas the residual market share is attributed to the “outside option” (fringe firms). Instead, the market for mobile phones in Greece was entirely covered by the three firms considered: Vodafone, Cosmote and Wind. Therefore, for this market the sum of the market shares of the firms considered is equal to one for each semester. Estimated market shares present the same characteristics.

**Fig. 3:**
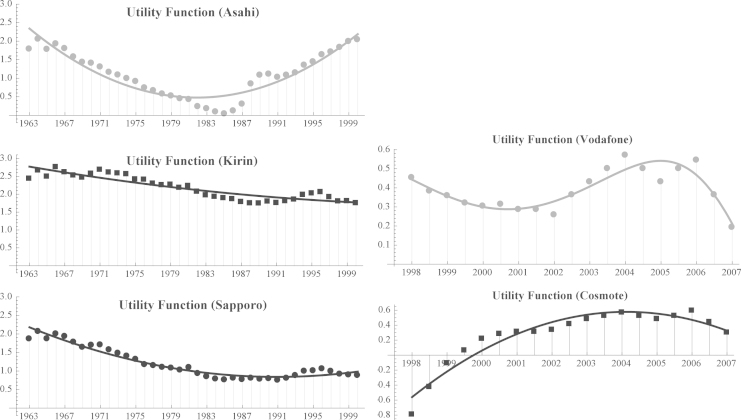
Utility functions and observed market shares of three Japanese beer companies (left panel), and of two mobile phone companies in Greece (right panel).

We describe how to obtain firms’ utility functions from historical data on market shares (see Fig. 3 and Eqs. (19)–(26) in [3]). This procedure also allows to estimate infra-annual market shares or to forecast future market shares (see Fig. 2 and refer to Supplementary materials). Last, we show how to use this procedure to identify firms’competitive roles from market shares in a few simple steps (see Table and Supplementary Figs. 1 and 2 in [3]).

### 2.2 Materials and methods

In [3] we introduced the following Lotka–Volterra model (LV) to describe market shares dynamics in a framework of competitive roles varying over time

(1) $$\frac{dx_{i}(t)}{\mathrm{dt}}=x_{i}\left(t\right)\left[g_{i}\left(t\right)-\sum_{j=1}^{N}g_{j}\left(t\right)x_{j}\left(t\right)\right],i=1,\cdots ,N.$$

If $x_{0}\left(t\right)$ denotes the market share of the *outside good*, then Eq. (1) describes the evolution of the market shares of all the inside goods.

In [3] and [4] it is shown that the analytical solutions of system (1) are

(2) $$x_{i}\left(t\right)=\frac{\exp(f_{i}(t))}{1+\sum_{j=1}^{N}\exp(f_{j}(t))},i=1,\ldots ,N,$$

where $f_{i}\left(t\right)$ is the *utility function* that a consumer assigns to the product of *i*-th firm, $g_{i}\left(t\right)={\dot{f}}_{i}(t)$ and $x_{i}\left(t_{0}\right)=\frac{\exp(f_{i}(t_{0}))}{1+\sum_{j=1}^{N}\exp(f_{j}(t))},\;i=1,\ldots ,N.$

Using Eq. (2), it is possible evaluate the utility functions $f_{i}\left(t\right)$ starting from the data on market shares. In fact, as in the classical logit model, we can easily obtain a set of the discrete values of the utility function as follows

(3) $$f_{i}\left(t\right)=\;\ln\left(x_{i}\left(t\right)\right)-\;\ln\left(x_{0}\left(t\right)\right),\;i=1,\cdots ,N$$

Using the routine FindFit of Mathematica® we find the time-dependent fit $f_{i}^{F}\left(t\right)$ for the utility functions $f_{i}\left(t\right),i=1,\cdots ,N$ (see the file Marasco et al. (nb format) in the Supplementary materials). In order to evaluate the fitting performance of the proposed model, we use the mean square error (MSE), the mean absolute percentage error (MAPE), and the fractional standard deviation (FS) to compare the historical data with the predicted values (see [5]). The error measures MSE, MAPE and FS can be calculated as follows (see the file mmc1 (nb format) in the Supplementary materials)

(4) $$\mathrm{MSE}=\frac{1}{n}\sum_{i=1}^{n}{(h_{i}-p_{i})}^{2},\mathrm{MAPE}=\frac{1}{n}\sum_{i=1}^{n}\left|\frac{h_{i}-p_{i}}{h_{i}}\right|100\%,\mathrm{FS}=2\frac{S_{h}-S_{p}}{S_{h}+S_{p}},$$

where $h_{i}$ and $p_{i}$ are respectively the historical and predicted values, and $S_{h}$ and $S_{p}$ are the standard deviations of historical and predicted values. The prediction capability levels of our model by means of MSE, MAPE, and FS are shown in [3, Table 3,7].

From the estimated utility functions ${f_{i}}^{F}\left(t\right)$ we determine a series of infra-annual (monthly) data of the market shares (see the files mmc4-5 in the Supplementary materials).

Finally, the estimated utility functions ${f_{i}}^{F}\left(t\right)$ allow us to establish the competitive roles of pairs of competitors as described in [3, Table 1] (see [6]).

The mathematical analysis presented in this brief work applies also to the case in which there is no outside good (see Eqs. (9), (11), (13) and (18) in [3]).

Tables 4 and 7 in [3] show that the utility functions accurately model the data on market shares in both cases.

Lastly, studying the signs of the functions *g_i_*(*t*) in Eq. (1), we note that in both case studies competing firms change their competitive roles over time. The competitive roles can be derived from the sign of the interaction coefficients according to Table 1.

### 2.3 Files description

• The file mmc2 (xls format) contains the annual market shares in the Japanese beer market. The file mmc3 (xls format) contains data on biannual market shares in the Greek mobile phone market.
• The file mmc4 (xls format) contains the estimated infra-annual market shares in the Japanese beer market. The file mmc5 (xls format) contains the estimated infra-annual market shares in the Greek mobile phone market.
• The file mmc1 (nb format) contains the code to derive the utility functions, the estimated market shares, the error functions (MSE, MAPE, and FS) to compare the historical data with the predicted values, and firm׳s competitive roles.

## Figures and Tables

**Table 1:** Competitive roles determined via interaction coefficients of three Japanese beer companies (left panel), and of three mobile phone companies in Greece (right panel).

**Time**	**Asahi** & *Kirin*	**Asahi** & *Sapporo*	**Kirin** & *Sapporo*
[1963,1982[	Mutualism	Mutualism	Mutualism
1982	Commensalism	Commensalism	Mutualism
]1982,1991[	Prey–predator	Prey–predator	Mutualism
1991	Prey–predator	Amensalism	Commensalism
]1991,1993]	Prey–predator	Pure competition	Predator–prey


**Time**	**Vodafone** & *Cosmote*		
[1998,2001[	Predator–prey		
2001	Amensalism		
]2001,2004[	Pure competition		
2004	Amensalism		
]2004,2005]	Prey–predator		
2005	Commensalism		
]2005,2007]	Mutualism		

## Footnotes

Appendix A Supplementary data associated with this article can be found in the online version at http://doi:10.1016/j.dib.2016.03.020.
