# Supplementary material for: Determining firms׳ utility functions and competitive roles from data on market shares using Lotka–Volterra models
Source: Data Brief. 2016 Mar 10;7:709–13. doi: 10.1016/j.dib.2016.03.020 (PMC4802538; doi:10.1016/j.dib.2016.03.020)
Supplement: Supplementary file 6 — Supplementary material [file mmc6.docx]

Conflicts of Interest Statement

Manuscript title: ***Determining firms' utility functions and competitive roles from data on market shares using Lotka-Volterra models***

The authors whose names are listed immediately below certify that they have NO affiliations with or involvement in any organization or entity with any financial interest (such as honoraria; educational grants; participation in speakers’ bureaus; membership, employment, consultancies, stock ownership, or other equity interest; and expert testimony or patent-licensing arrangements), or non-financial interest (such as personal or professional relationships, affiliations, knowledge or beliefs) in the subject matter or materials discussed in this manuscript.

Author names:

Addolorata Marasco, Antonella Picucci, Alessandro Romano
